# Identification of the Imprinted *KLF14* Transcription Factor Undergoing Human-Specific Accelerated Evolution 

**DOI:** 10.1371/journal.pgen.0030065

**Published:** 2007-05-04

**Authors:** Layla Parker-Katiraee, Andrew R Carson, Takahiro Yamada, Philippe Arnaud, Robert Feil, Sayeda N Abu-Amero, Gudrun E Moore, Masahiro Kaneda, George H Perry, Anne C Stone, Charles Lee, Makiko Meguro-Horike, Hiroyuki Sasaki, Keiko Kobayashi, Kazuhiko Nakabayashi, Stephen W Scherer

**Affiliations:** 1 Program in Genetics and Genomic Biology, The Hospital for Sick Children, Toronto, Ontario, Canada; 2 The Centre for Applied Genomics, The Hospital for Sick Children, Toronto, Ontario, Canada; 3 Department of Molecular and Medical Genetics, University of Toronto, Toronto, Ontario, Canada; 4 Institute of Molecular Genetics (IGMM), CNRS UMR5535, Montpellier, France; 5 University of Montpellier II, Montpellier, France; 6 Institute of Child Health, University College London, London, United Kingdom; 7 Division of Human Genetics, Department of Integrated Genetics, National Institute of Genetics, Research Organization of Information and Systems, Mishima, Japan; 8 School of Human Evolution and Social Change, Arizona State University, Tempe, Arizona, United States of America; 9 Department of Pathology, Brigham and Women's Hospital, Boston, Massachusetts, United States of America; 10 Department of Genetics, School of Life Science, The Graduate University for Advanced Studies (Sokendai), Mishima, Japan; 11 Department of Molecular Metabolism and Biochemical Genetics, Kagoshima University Graduate School of Medical and Dental Sciences, Kagoshima, Japan; Santa Fe Institute, United States of America

## Abstract

Imprinted genes are expressed in a parent-of-origin manner and are located in clusters throughout the genome. Aberrations in the expression of imprinted genes on human Chromosome 7 have been suggested to play a role in the etiologies of Russell-Silver Syndrome and autism. We describe the imprinting of *KLF14*, an intronless member of the Krüppel-like family of transcription factors located at Chromosome 7q32. We show that it has monoallelic maternal expression in all embryonic and extra-embryonic tissues studied, in both human and mouse. We examine epigenetic modifications in the *KLF14* CpG island in both species and find this region to be hypomethylated. In addition, we perform chromatin immunoprecipitation and find that the murine *Klf14* CpG island lacks allele-specific histone modifications. Despite the absence of these defining features, our analysis of *Klf14* in offspring from *DNA methyltransferase 3a* conditional knockout mice reveals that the gene's expression is dependent upon a maternally methylated region. Due to the intronless nature of *Klf14* and its homology to *Klf16*, we suggest that the gene is an ancient retrotransposed copy of *Klf16*. By sequence analysis of numerous species, we place the timing of this event after the divergence of Marsupialia, yet prior to the divergence of the Xenarthra superclade. We identify a large number of sequence variants in *KLF14* and, using several measures of diversity, we determine that there is greater variability in the human lineage with a significantly increased number of nonsynonymous changes, suggesting human-specific accelerated evolution. Thus, *KLF14* may be the first example of an imprinted transcript undergoing accelerated evolution in the human lineage.

## Author Summary

Imprinted genes are expressed in a parent-of-origin manner, where one of the two inherited copies of the imprinted gene is silenced. Aberrations in the expression of these genes, which generally regulate growth, are associated with various developmental disorders, emphasizing the importance of their discovery and analysis. In this study, we identify a novel imprinted gene, named *KLF14*, on human Chromosome 7. It is predicted to bind DNA and regulate transcription and was shown to be expressed from the maternally inherited chromosome in all human and mouse tissues examined. Surprisingly, we did not identify molecular signatures generally associated with imprinted regions, such as DNA methylation. Additionally, the identification of numerous DNA sequence variants led to an in-depth analysis of the gene's evolution. It was determined that there is greater variability in *KLF14* in the human lineage, when compared to other primates, with a significantly increased number of polymorphisms encoding for changes at the protein level, suggesting human-specific accelerated evolution. As the first example of an imprinted transcript undergoing accelerated evolution in the human lineage, we propose that the accumulation of polymorphisms in *KLF14* may be aided by the silencing of the inactive allele, allowing for stronger selection.

## Introduction

Genomic imprinting is an epigenetic phenomenon characterized by the expression of alleles in a parent-of-origin manner, giving rise to monoallelic or heavily biased gene expression. Imprinted genes are generally located in clusters that often contain maternally and paternally expressed protein-coding genes, as well as imprinted noncoding transcripts [1]. Aberrations in the expression of imprinted genes have been associated with various developmental and behavioral disorders, such as Prader-Willi syndrome and Beckwith-Wiedemann syndrome.

Imprinted genes on human Chromosome 7 have been suggested to underlie several disorders that show parent-of-origin effects, including Russell-Silver Syndrome (RSS). This genetically heterogeneous disorder, which is characterized by intrauterine and postnatal growth restriction as well as dysmorphic facial features, has been associated with numerous chromosomal rearrangements and anomalies. Most recently, hypomethylation of the imprinting control region (ICR) at Chromosome 11p15 has been associated with RSS [2]. However, various reports have suggested a possible role for human Chromosome 7 in the etiology of this disorder, based on evidence indicating that 10% of affected individuals have maternal uniparental disomy for this chromosome [3,4]. A causative gene for the Chromosome-7 form of RSS has not been found, but absence of a paternally inherited *FOXP2* gene might explain the verbal dyspraxia phenotype usually observed in this subtype [5]. A recent study has also shown evidence for the existence of a parent-of-origin effect in autism linked to Chromosome 7 [6]. As a consequence, studies that have attempted to identify imprinted genes associated with these disorders have concentrated on Chromosome 7 [7,8].

**Figure 1:**
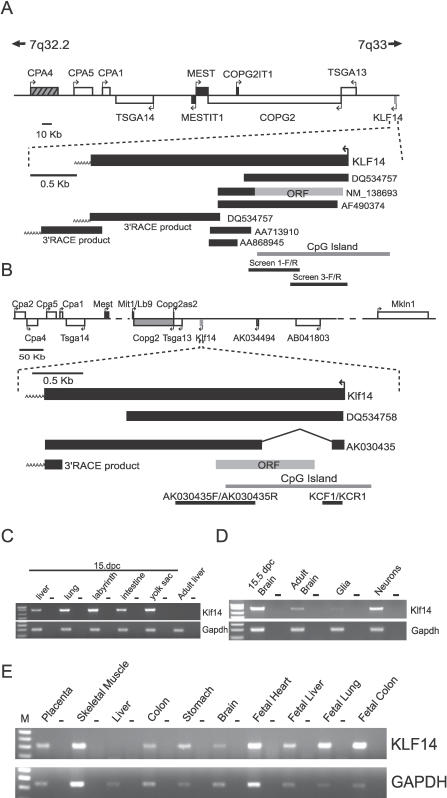
Human and Murine *KLF14* Structure and Expression (A) Human *KLF14* and (B) murine *Klf14* structure are shown. Genes in 7q32.3 region in the upper panel with maternally and paternally expressed genes are depicted in grey and black, respectively. Striped patterns represent genes with tissue specific imprinting. Arrows indicate transcriptional direction. Lower panels show the gene structure of *KLF14*/*Klf14*, including results from RACE, the ORF, CpG island, and primers used in various analyses (represented by thin black bars). The grey block representing AK030435 denotes the fact that evidence of splicing was not identified in our experiments. (C) Expression of *Klf14* in murine embryonic and extra-embryonic and (D) brain tissues is shown. Samples lacking reverse transcriptase are indicated by -. Results indicate higher levels of expression in extra-embryonic tissues. (E) Expression of *KLF14* in human tissues. Human expression results concur with those of mouse expression, in that there is higher expression in prenatal stages of development.

To date, three distinct imprinted loci have been identified on human Chromosome 7. The first, located at 7p12.2, contains the growth factor receptor-bound protein 10 gene (*GRB10*) [9]. The second locus, at 7q21.3, includes the retrotransposon derived *PEG10* [10] and ɛ-sarcoglycan (*SGCE*) [11]. The third cluster, located at 7q32.3, includes the genes encoding carboxypeptidase-A4 (*CPA4*) [12] and mesoderm specific transcript homolog (*MEST*) [13] (Figure 1). Hannula and colleagues described a patient with RSS with partial maternal uniparental disomy for 7q31-qter, highlighting the genes in this third region as candidates for the syndrome [5,14]. However, analyses of imprinted transcripts in this interval have only excluded them as candidates for RSS [15,16]. Several studies have attempted to identify additional imprinted transcripts in the region, but have not found parent-of-origin specific expression in tissues analyzed [17,18].

In this paper, we describe the identification of a novel maternally expressed imprinted transcript located at 7q32.3, telomeric to *TSGA13*. This gene, named *KLF14* (Krüppel-like factor 14), is an intronless molecule and a member of the Sp/KLF family of transcription factors. These proteins are characterized by three highly conserved C2H2-type zinc-fingers at the carboxy-terminal end joined to each other by linker sequences, known as Krüppel-links [19]. In contrast, the N terminus is highly variable between KLF paralogues and has lower levels of conservation between orthologues [20]. Members of the KLF family are known to act as transcriptional activators, repressors, or both [21].

We show that *KLF14* has monoallelic maternal expression in a variety of embryonic and extra-embryonic tissues in human and mouse. In addition, we determine that *KLF14* has undergone accelerated evolution in the human lineage with numerous amino acid substitutions identified in different populations and demonstrate that this variability is increased in the human lineage.

## Results

### Imprinting Analyses

#### Maternal specific expression of human and murine *KLF14* in embryonic and extra-embryonic murine tissues.

**Figure 2:**
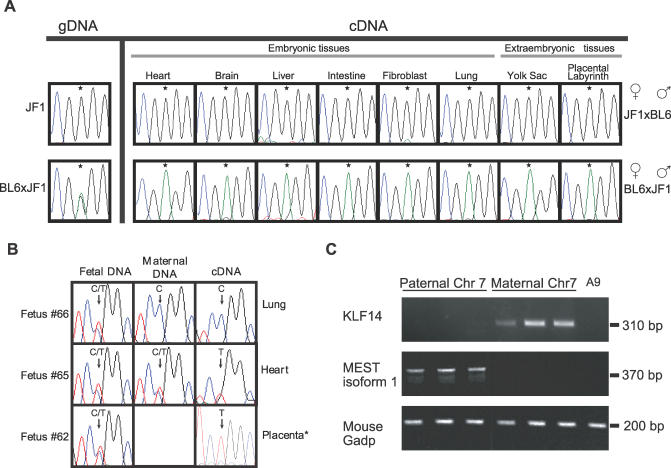
Imprinting Analysis of Human and Murine *KLF14* (A) Imprinted expression of murine *Klf14* is shown. Sequence analysis of genomic DNA and RT-PCR products from 15.5-dpc hybrid mice are shown in the left and right panels, respectively. Genomic sequencing results indicate the genotype for JF1 (G) at the polymorphism. RT-PCR sequencing results show the expression of the JF1 allele in all tissues where JF1 is the maternal allele (upper row in right panel) and expression of the BL6 allele in the reciprocal cross (lower row in right panel), indicating maternal expression. (B) Imprinted expression of human *KLF14*. The first column of panels shows genomic sequencing electropherograms for three fetal samples (rows) heterozygous for a polymorphism in *KLF14*. The second column presents the genotype for the corresponding maternal samples (maternal DNA was not available for fetus number 62). The third column shows sequencing results of RT-PCR products indicating the monoallelic expression of various tissues, as indicated on the right of the column. Results from fetus number 66, which is informative for parental origin, indicate that *KLF14* is maternally expressed. *, sequencing of tongue, stomach, eye, kidney, and intestine cDNA from fetus number 62 showed monoallelic expression. (C) Maternal expression of human *KLF14* in somatic cell hybrids. RT-PCR was performed for three independent maternal or paternal monochromosomal hybrid cell lines for human Chromosome 7. Results confirm the maternal expression of *KLF14*, as seen in (B). The expression of the paternally expressed *MEST* and mouse A9 cell line, which lacks human Chromosome 7, are also shown.

To determine the allelic expression of murine *Klf14*, reciprocal crosses of C57BL/6J (BL6) and JF1/Ms (JF1) were carried out and cDNA was extracted from embryonic and extra-embryonic tissues at 15.5 days post coitum (dpc). To distinguish the two parental alleles, a G/A polymorphism corresponding to nucleotide 451 of AK030435 was identified. Equal peak heights of the two alleles were observed in sequencing electropherograms from PCR-amplified genomic DNA of BL6 × JF1 *F*_1_ hybrids, indicating lack of amplification bias (Figure 2A). cDNA from the *F*_1_ hybrids was amplified, the products were sequenced, and allelic expression was analyzed in an intronless PCR fragment amplified by primers AK030435F/AK030435R. Due to the intronless nature of the amplicon, samples without reverse transcriptase were also prepared to account for the possibility of genomic DNA contamination. By use of reciprocal crosses of hybrid mice, monoallelic expression of the maternal allele was identified in all tissues examined, as noted by the expression of a single peak at the position corresponding to the G/A polymorphism (Figure 2A). In addition, *Klf14* was found to be imprinted in tissues extracted from 9.5 dpc embryos and neonates (unpublished data), indicating that the imprinted expression of *Klf14* is not developmental-stage specific and is an imprint established early in development.

To distinguish parental alleles of human *KLF14,* DNA derived from fetal samples was genotyped for a C/T polymorphism at nucleotide 336 of NM_138693, and three fetuses heterozygous for the polymorphism were identified (Figure 2B). cDNA corresponding to *KLF14* was sequenced, and the expression of the alleles was noted. Monoallelic expression of *KLF14* was observed in lung (fetus number 66), heart (fetus number 65), tongue, stomach, eye, intestine, and placental samples (fetus number 62). One informative fetus-mother DNA pair indicated monoallelic expression of the maternal allele (fetus number 66).

We examined the expression of *KLF14* in cDNA extracted from somatic cell hybrid lines containing a maternal or paternal human Chromosome 7. These lines have been shown to maintain the monoallelic expression of *MEST-isoform-1* and *MESTIT1* [22]. PCR was carried out, amplifying a fragment specific to *KLF14*. The absence of a PCR product in a cell line not containing the human chromosome (A9) indicated that the amplification was specific to a human Chromosome-7 transcript. Amplification of *KLF14* was observed exclusively in cell lines containing a maternal human Chromosome 7 (Figure 2C), indicating maternal specific expression.

#### Histone modifications in *Klf14* and *Mest* CpG islands.

The allele-specific modification of histones has been shown to be a hallmark of promoters and ICRs of imprinted loci [23,24]. Though such modifications are a feature of preferential expression when found in the promoter, they are integral at the level of ICRs. The unmethylated allele of ICRs is associated with “open chromatin” marks, such as the acetylation of histones and dimethylation of lysine 4 on histone 3 (H3K4me2). In contrast, the methylated allele of ICRs is characterized by heterochromatic features, such as hypoacetylation of lysine 9 on histone 3 (H3K9ac), trimethylation of lysine 9 on histone 3 (H3K9me3), and trimethylation of lysine 20 on histone 4 (H4K20me3) [25,26]. Thus, by performing chromatin immunoprecipitation (ChIP), we investigated allele-specific covalent histone modifications that have been shown to be characteristic of maternally and paternally methylated ICRs [23,24,27,28] (P. Arnaud and R. Feil, personal communication).

**Figure 3:**
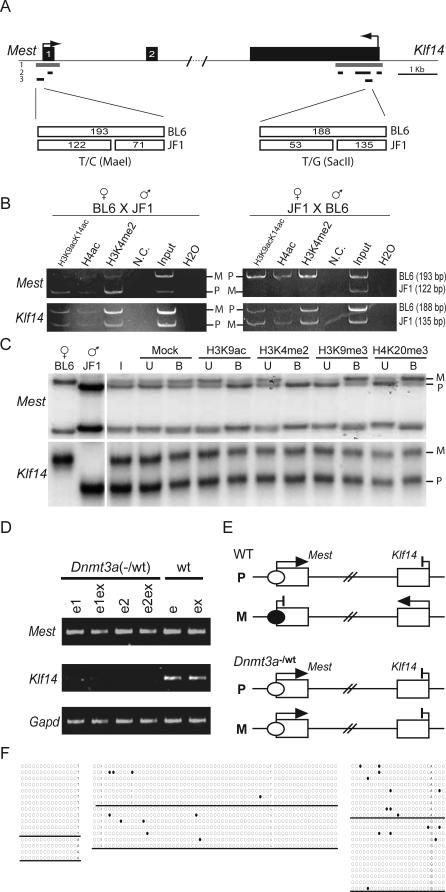
Epigenetic Modifications of Murine *Klf14* (A) The location and distribution of regions analyzed in *Mest* and *Klf14*. The CpG islands overlapping *Mest* exon 1 and *Klf14* are depicted by grey bars (row 1). The regions examined in the methylation analysis and ChIP assay are indicated in rows 2 and 3, respectively. The restriction enzymes used in the ChIP assay and the polymorphisms identified in BL6 and JF1 strains are also shown. (B) Analysis of histone modifications by ChIP in fibroblast cells of BL6 × JF1 and JF1 × BL6 hybrids. ChIP was performed using formaldehyde fixed chromatin. Antibodies against histone 3 acetylated at lysines 9 and 14 (H3K9acK14ac), histone 4 acetylated at lysines 5, 8, 12, and 16 (H4ac), and H3K4me2 were used in the ChIP assay. Precipitated DNAs were PCR amplified using primers specific to the CpG islands of *Mest* and *Klf14* and subsequently digested as shown in (A). DNA before immunoprecipitation (input) and the product obtained with no antibody (N.C.) were also included in the analysis. The difference in band intensities between the precipitated products and input DNA reveals that there is preferential precipitation of H3K9acK14ac, H4ac, and H3K4me2 on the paternal allele of the *Mest* CpG island, but no allelic differences were detected at the *Klf14* region. (C) Analysis of histone modifications by native-ChIP in whole embryos of BL6 × JF1 hybrids. Chromatin was immunoprecipitated using antibodies against H3K9ac, H3K4me2, H3K9me3, and H4K20me3. Anti-chicken was used as a nonspecific antibody (mock). Input DNA is denoted by I. Antibody-bound and unbound fractions of the precipitate are denoted by B and U, respectively. Precipitated DNA was PCR amplified using the same primers as in (B). The amplified DNA was analyzed by single strand conformation polymorphism. The results show differences in histone modifications between the two parental alleles in the *Mest* CpG island, but allelic enrichments were not observed for *Klf14*. (D) Expression of *Klf14* in offspring of *Dnmt3a* conditional knockout mice. The expression of *Mest* and *Klf14* was examined in two embryos (e1–2) and corresponding extra-embryonic tissues (e1-2ex) from the offspring of female *Dnmt3a* conditional knockout mice, as well as a wild-type (wt) embryo. *Klf14* expression is lost in the knockout mice, suggesting that its expression is dependent upon a maternally methylated region. (E) Model of *Klf14* expression in wild-type (wt) and *Dnmt3a* conditional knockout mice. In wild-type mice (upper panel), the maternally methylated CpG island in *Mest* (black circle) silences the expression of the gene from the maternal allele (M), while *Klf14* is actively transcribed on this allele. The opposite pattern of expression is seen on the paternal strand (P), because of the unmethylated CpG island (white circle). In *Dnmt3a* −/+ embryos (lower panel), maternal methylation of the *Mest* CpG island is lost, causing increased expression of *Mest* and loss of expression of *Klf14*. (F) Bisulfite sequencing results from 12.5-dpc whole embryos of BL6 × JF1 hybrids. Each block corresponds to a separate region analyzed in the *Klf14* CpG island, as shown in (A) (*Mest* methylation analysis is not shown). Hollow circles and black circles indicate unmethylated and methylated CpG dinucleotides, respectively (N, could not be determined). Each row of circles represents CpGs in an individual PCR product clone. In each block, the top section and bottom sections correspond to clones from the maternal allele (BL6) and paternal (JF1) alleles, respectively, as determined by use of polymorphisms. The three regions analyzed indicate that the *Klf14* CpG island is hypomethylated on both alleles.

We performed ChIP by formaldehyde fixation using murine fibroblasts from the *F*_1_ hybrid offspring of BL6 × JF1. We previously determined that *Klf14* is imprinted in these cells (Figure 2A), and therefore epigenetic modifications associated with the gene would be present. To distinguish each allele, we identified single nucleotide polymorphisms (SNPs) in the CpG islands of murine *Mest* and *Klf14,* which were also restriction fragment length polymorphisms of *Mae*I and *Sac*II (Figure 3A). Although histone 3 acetylation (at lysines 9 and 14), histone 4 acetylation (at lysines 5, 8, 12, and 16), and H3K4me2 were exclusively enriched in nucleosomes of the unmethylated paternal allele of *Mest,* no differences were observed between the parental alleles at *Klf14* (Figure 3B).

We subsequently performed native ChIP, which precipitates a higher quantity of chromatin in comparison to formaldehyde fixed chromatin [29], to determine if a more sensitive assay would identify differences in histone modifications in the CpG island of *Klf14*. For this analysis we used DNA from the 13.5-dpc whole embryos of hybrid BL6 × JF1 mice. Precipitated DNA was PCR amplified and analyzed by the single strand conformation polymorphism method (Figure 3C). Again, we determined that H3K9ac and H3K4me2 were associated with the unmethylated paternal allele in the *Mest* promoter, and that H3K9me3 and H4K20me3 were associated with the methylated maternal allele. However, we did not observe differences between the alleles of *Klf14*.

#### Methylation analysis and *Klf14* expression in *DNA methyltransferase 3a* knockout mice.

The establishment of a differentially methylated region (DMR) in the germ-line is the primary hallmark of imprinted loci and has been shown to be necessary for the proper imprinting of many regions (for review, see [30]). The study of these regions has been greatly enhanced by *DNA methyltransferase 3a (Dnmt3a)* conditional knockout mice. These mice have *Dnmt3a* disrupted specifically in germ cells, while somatic cells express the wild-type protein, allowing conditional knockouts to be viable and enabling the study of their offspring. The progeny of female conditional knockouts die in utero at approximately 10.5 dpc and have been shown to be hypomethylated at maternally methylated DMRs, while methylation patterns at paternally methylated regions and repetitive regions remain unaltered [31]. Correspondingly, a 1.6-fold increase in the expression of *Mest* has been measured in these *Dnmt3a^−^* ^/wt^ embryos indicating a relaxation of the imprinted expression of the gene. We examined the expression of *Mest* and *Klf14* in the offspring of female *Dnmt3a* conditional knockout mice. Our results indicate a substantial decrease in *Klf14* expression, in both embryonic and extra-embryonic tissues in *Dnmt3a^−^* ^/wt^ embryos (Figure 3D). This suggests that *Klf14* expression is dependent upon the establishment of maternal imprints in oocytes (Figure 3E).

We examined the methylation status of the CpG island located in *Klf14*. Using three different PCR primers spanning the CpG island (Figure 3A), we subcloned PCR-amplified bisulfite-treated DNA extracted from 12.5-dpc BL6 × JF1 hybrids. We observed hypomethylation of both alleles throughout the CpG island (Figure 3F). Similar results were obtained from the bisulfite sequencing of fibroblast DNA (unpublished data). Using the same materials, we examined the methylation of *Mest* and observed enrichment of methylated CpGs on the maternal allele, as previously described [32] (unpublished data).

The methylation status of 94 CpG dinucleotides located in the open reading frame (ORF) and 5′ UTR regions of human *KLF14* were examined by bisulfite sequencing of fibroblast DNA. Extensive hypomethylation was again observed in the subcloned fragments (unpublished data).

### *Klf14* Structure and Function

#### Characterization of the human and murine *KLF14* transcripts.

An in silico analysis of human *KLF14* was performed to identify the full-length transcript of the gene by EST assembly. A single intronless transcript of approximately 1.4 Kb was found in the Chromosome 7 annotation database (http://www.chr7.org) [33]. This reference sequence, derived from mRNA AF490374, contains an ORF of 972 nucleotides, as well as an in-frame stop codon. Rapid amplification of cDNA ends (RACE) was performed, and single band of approximately 1.6 Kb was amplified and directly sequenced (Figure 1A). The unspliced fragment contained a poly-A tail and poly-A signal. We performed 3′ RACE again using primers located closer to the new 3′ end, and a second fragment, also containing a poly-A tail, was identified.

In silico analysis was performed to identify the full length of murine *Klf14*. The spliced transcript AK030435, whose putative second exon was originally used to determine the imprinted expression of the gene, contains a poly-A signal. Reverse transcriptase-PCR (RT-PCR) was performed to confirm the expression of the spliced transcript, yet such attempts only succeeded in amplifying cDNA intronic to AK030435 and did not find evidence of splicing. An ORF of 978 nucleotides was identified, partially located in the intron of AK030435, corresponding to *Klf14* (Figure 1B). We performed 3′ RACE, and the results did not extend the transcript beyond the 3′ end of AK030435, though they identified a poly-A tail.

#### Expression of human and murine *KLF14.*

To determine the expression of *KLF14* in human tissues, RT-PCR was carried out using numerous adult and fetal RNA samples. In general, the transcript was found to have low levels of expression in both human and mouse. The transcript was found to be expressed in many tissues (Figure 1E), but its expression was absent in liver and lymphoblast (unpublished data). It was found to have higher levels of expression in fetal tissues and placenta than in adult tissues.

RT-PCR was performed on murine cDNA, where higher levels of *Klf14* expression were observed in embryonic and extra-embryonic tissues with respect to adult tissues (Figure 1C). We cultured glia and neurons from mouse embryos, as previously described [34], and observed much higher level of expression in the latter (Figure 1D).

#### Syntenic analysis of *KLF14.*

The intronless nature of *KLF14* suggested that the gene may have arisen through retrotransposition [35]. Phylogenetic studies of the KLF family have revealed that the KLF14 protein is most closely related to KLF16, encoded on human Chromosome 19 [36]. An amino acid alignment of these two proteins (BLASTP) demonstrated that they are 58% identical and bear most similarity in the first 26 amino acids (N terminus) and the zinc-finger domains (C-terminal end). Thus, it is plausible that this gene is an ancient retrotransposon-derived duplication of *KLF16*.

**Figure 4:**
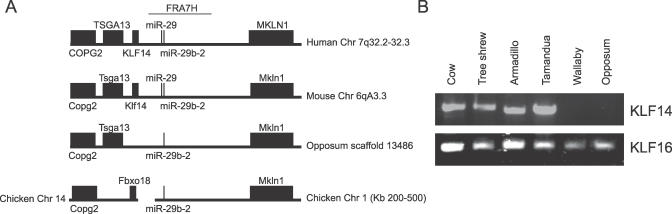
Retrotransposition of *KLF14* and Mammalian Evolution (A) Genomic distribution of *KLF14* and flanking genes is shown. *KLF14* is flanked by *MKLN1* and *TSGA13* in human and mouse. These two genes are present in opossum, yet *KLF14* is absent. In chicken, there is a syntenic break in the region, placing genes on different chromosomes. Two microRNAs (miR-29 and miR-29b-2), which lie in the fragile site adjacent to *KLF14* (FRA7H), are conserved. (B) Presence of *KLF14* and *KLF16* in distant mammals is shown. The lower panel shows PCR amplification of *KLF16* from mammals of diverse clades, and the upper panel shows the amplification of *KLF14*. The mammals shown are (L-R) cow *(Bos taurus),* tree shrew *(Tupaia glis),* nine-banded armadillo *(Dasypus novemcinctus),* tamandua *(Tamandua tetradactyla),* red-necked wallaby *(M. rufogriseus),* and red-legged short-tailed opossum *(M. brevicaudata).* It indicates that *KLF14* is present in eutherian, but not marsupial mammals, and shows that *KLF16* is more ancient than *KLF14*. 10.1371/journal.pgen.0030065.g004

To determine the timing of the retrotransposition event during vertebrae evolution, we examined the synteny of the region encompassing *Klf14* in human, mouse, opossum, and chicken using the genome browser at University of California, Santa Cruz (http://genome.ucsc.edu). We used *COPG2*/*TSGA13* and *MKLN1* as reference points, which are located centromeric and telomeric to human *KLF14,* respectively. Two highly conserved segments corresponding to microRNAs (miR-29 and miR-29b-2) were also used as anchors. Synteny with all these elements, including *Klf14*, was maintained in mouse (Figure 4A). However, *Klf14* and miR-29 were absent in the opossum. A break in synteny was observed in chicken, where *Copg2* and *Mkln1* were located on different chromosome. This analysis suggested that *Klf14* was retrotransposed after the divergence of marsupials from eutherian mammals.

A more precise timing of this retrotransposition event in eutherian evolution was examined by amplifying *Klf14* in organisms from each of the supraordinal clades: Afrotheria, Xenarthra, Euarchontoglires, and Laurasiatheria [37]. Using a variety of different PCR conditions, *Klf14* was amplified from numerous species and its sequence was confirmed by direct sequencing of products. Several primers were designed, located in the conserved zinc-finger domains of the gene. Multiple attempts using increasingly more permissive conditions were used to amplify DNA from red-legged short-tailed opossum *(Monodelphis brevicaudata),* red-necked wallaby *(Macropus rufogriseus),* and echidna *(Tachyglossus aculeatus),* yet bands corresponding to a *Klf14* homologue were not obtained. However, *Klf16* was amplified in both eutherian and marsupial mammals (Figure 4B).

The syntenic analysis together with the amplification of *Klf14* in each of the superclades, most notably in members of the Xenarthra order (represented by the armadillo and tamandua), suggest that the gene is present in all eutherian mammals, yet absent in monotremes and marsupials. Based on estimates of mammalian evolution, this would place the retrotransposition event, which gave rise to *KLF14,* between 130 and 170 million years ago (i.e., prior to the divergence of Xenarthra and after the divergence of Marsupialia) [38]. At the same time, the presence of *Klf16* in marsupials indicates that it is more ancient than *KLF14* and supports our hypothesis that the latter gene may be a retrotransposed copy of *KLF16*.

### *KLF14* Variation and Selection

#### Sequence of *KLF14* in RSS and autistic individuals.

As previously mentioned, imprinted genes on Chromosome 7 are hypothesized to play a role in the etiologies of RSS and autism. The *KLF14* ORF was sequenced in 60 RSS patients and 160 autistic individuals to test for mutations. Although, we did not identify mutations specific to these affected groups, we identified numerous nonsynonymous base-pair substitutions in the N-terminal region (Figure S1). All polymorphic changes were found at equal frequencies in controls, suggesting that they are not disease-associated.

**Figure 5:**
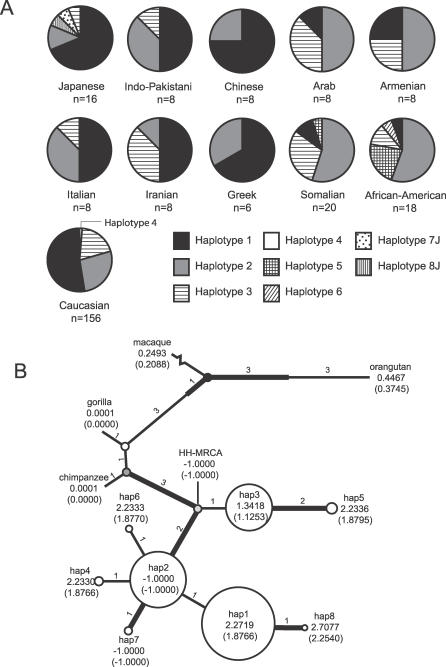
Haplotype Frequencies of *KLF14* ORF in the Human Population (A) The frequency of eight *KLF14* haplotypes in various ethnic populations is shown. The frequency of each haplotype, as defined in Figure S1, identified in ethnic populations is shown (*n* = number of chromosomes genotyped). (B) *KLF14* primate species and human haplotype tree is represented. This tree was created manually by parsimony, with the inferred number of changes shown on each branch (thick lines represent nonsynonymous changes, while thin lines represent synonymous changes). The tree is rooted using two macaque sequences (M. mulatta and M. nemestrina). In orang-utan, gorilla, and chimpanzee, only a single haplotype is represented (where polymorphisms were present, the ancestral allele was used; when descendent alleles were used for polymorphisms, the results did not differ significantly). MRCAs, manually inferred by parsimony, are represented by shaded circles at four nodes in the tree. dN/dS values were calculated for each lineage, including the fixed human sequence (HH-MRCA) and each of the human haplotypes. For the human haplotypes, the dN/dS value is calculated to the human-chimpanzee MRCA, and not to the HH-MRCA. Two methods were utilized: maximum likelihood pairwise comparison (top) and Nei and Gojobori comparison (bottom). Where dS = 0, these methods give values −1.0000. 10.1371/journal.pgen.0030065.g005

The *KLF14* ORF was sequenced in a total of 704 chromosomes, representing both patients and controls, the latter of which included an ethnically diverse panel of 61 individuals. We found eight haplotypes (Figure S1): two were specific to the Japanese population (haplotypes 7 and 8), and one was found predominantly in individuals of African descent (haplotype 6) (Figure 5A). The frequency of these haplotypes varied greatly in different populations, suggesting that the gene may have recently undergone relaxed selection or accelerated evolution.

#### Lineage-specific accelerated evolution of *KLF14.*

To analyze evolutionary features of *KLF14*, we sequenced its ORF in three gorillas, two orang-utans, two macaques, two bonobos, and 20 chimpanzees (for genotypes, see Tables S4 and S5). The ratio of nonsynonymous to synonymous substitutions (dN/dS) was calculated in phylogenetic analysis by maximum likelihood (PAML) software [39] using a two-ratio maximum-likelihood analysis between the human haplotype's most recent common ancestor (HH-MRCA) and the human-chimpanzee MRCA (Figure 5B). This measurement, which only evaluates fixed changes, is used to determine selective pressures in a coding region, where values >1 suggest positive selection, while values <1 indicate purifying selection. This dN/dS value was found to be −1.0000 (or infinity), because no synonymous changes occurred in this lineage.

We also calculated dN/dS values for each of the eight human haplotypes from the human-chimpanzee MRCA (Figure 5B). In each of these haplotypes, the dN/dS value was >1, with the two most common haplotypes (1 and 2) having values of 2.25 and −1.0000 (or infinity), respectively. Such values suggest that positive selection has occurred in the human lineage. Consequently, we used the likelihood ratio test within PAML [39] for detecting positive selection and found that the dN/dS from the HH-MRCA was not significantly >1 (*p* = 0.1345) (Table S1). Therefore, we could not reject the hypothesis of neutral evolution in the human lineage. This conclusion was confirmed by analyzing the dN/dS values for each of the human haplotypes. Only one haplotype (7) had a dN/dS value significantly >1 (*p* < 0.05) before Bonferroni multiple test correction. However, this is a rare haplotype, and after Bonferroni correction its significance is lost. The common haplotypes (1 and 2) did not have dN/dS values significantly >1. Similar results were obtained when all human haplotypes were simultaneously used to identify significant elevation in the human dN/dS (Text S1).

By sequencing regions flanking the ORF of *KLF14*, we attempted to find evidence of positive selection in the form of a recent selective sweep. We sequenced 9,880 bps, including the ORF, in 18 individuals from our diversity panel. A chimpanzee was also sequenced to identify the ancestral allele for each variant. In total, 42 SNPs and two polymorphic insertion/deletions were identified, 13 of which were singletons, and 29 of which were parsimony-informative sites. Using DnaSP 4.0 [40], we performed various analyses including Tajima's D-test [41], Fu and Li's D-test [42], Fu and Li's F* [42], Fu's Fs statistic [43], and Fay and Wu's H test statistic [44]. These tests are designed to detect recent selective sweeps by looking for skews in frequency spectrums or recent mutations relative to ancient mutations. Departures from neutrality detected by these tests could indicate that positive selection has occurred. However, none of these analyses gave a significant result.

The McDonald-Kreitman test [45], which examines ratios of synonymous and nonsynonymous nucleotide substitutions within and between species, was performed using the human ORF sequences compared to those from the 20 chimpanzee and two bonobo samples. Again, this test did not give a significant result. These findings, together with the results from the previous analyses, indicate that we cannot reject the null hypothesis of neutrality in *KLF14*. Although there is evidence suggesting that the gene may be undergoing positive selection, such as the presence of three fixed nonsynonymous changes (bps 194, 545, and 559) and two nonsynonymous SNPs (bps 140 and 172) for which the derived allele is more frequent than the ancestral allele, our results may also be explained by accelerated neutral evolution.

To establish whether accelerated evolution has ocurred, dN/dS values were calculated for the sequenced primates with respect to their MRCAs. In each case, the dN/dS values were <1, indicating purifying selection (Figure 5B). Subsequently, we examined whether the dN/dS values observed in the human lineage were significantly elevated relative to the primate background dN/dS ratio by using the likelihood ratio test [39]. For this analysis, the dN/dS value for HH-MRCA showed significance (*p* = 0.0139). Additionally, all haplotypes with the exception of haplotype 3 had significantly increased dN/dS values (*p* < 0.05) before Bonferroni correction. After Bonferroni correction, only haplotypes 2 and 7 remained significant (see Table S1). We confirmed the significantly higher dN/dS values in the human lineage relative to the primate background using the haplotype tree (see Text S1). Significant results were obtained for the human lineage using both the two- and three-ratio tests in PAML. These results suggest accelerated evolution in the human lineage.

We used a binomial distribution [46] to determine if there is an increased rate of amino acid substitutions in the human lineage relative to the ape lineages (orang-utan, gorilla, and chimpanzee). The results indicated that seven of the eight haplotypes were significant before and after Bonferroni correction (*p* < 0.05), with the exception of haplotype 3 (*p* = 0.0540) (Table S2). Yet when this test was applied to the HH-MRCA, a significant result was not obtained (*p* = 0.0540). However, the significance observed in the seven haplotypes suggests that, although there is no significant evidence for positive selection, there is human lineage-specific accelerated protein evolution of *KLF14*.

#### Sequence variability in *KLF14.*

To determine if the sequence variability observed in *KLF14* was significantly greater than average, we used information available from the Seattle SNP (http://pga.gs.washington.edu) and Environmental Genome (http://www.niehs.nih.gov/envgenom/home.htm) projects to extract polymorphism data from 826 genes [47,48]. These genes were sequenced in >96 haploid genomes of ethnically diverse individuals, allowing for the identification of 99.9% of SNPs with minor allele frequencies ≥5% [49]. Using the allele frequencies of identified polymorphisms, we determined π values for each of the sequenced genes and for *KLF14*. π is the measure of nucleotide diversity or the average number of pairwise nucleotide differences between aligned sequences divided by the sequence length [50]. We found that *KLF14* has a higher π value than 798 of the 827 total genes (96.5%). We then calculated π values at nonsynonymous and synonymous SNP sites separately and determined that *KLF14* had a higher π value than 779 of the 827 genes (94.2%) within each of these sets, revealing that the gene's significant enrichment in SNPs is due to elevated numbers of both synonymous and nonsynonymous polymorphisms.

To determine if the degree of variability observed is unique to human *KLF14* or is common in other primates, polymorphisms in the ORF were identified in 20 chimpanzees. Only three SNPs were observed, all of which were synonymous singleton changes. We found two of these variations in a single chimpanzee of the subspecies *Pan troglodytes schweinfurthii*. In addition, sequence comparison between two bonobos *(P. paniscus)* and the chimpanzees did not identify any polymorphisms. To look for increased diversity in the human lineage, we calculated diversity statistics (π and θ) for both synonymous and nonsynonymous sites using non-patient human samples (264 chromosomes) and chimpanzees (40 chromosomes) (Table S3). θ is a measure of genetic diversity within populations that describes the amount of variation expected at each nucleotide site [50]. In general, π and θ values were higher in the human gene than in the chimpanzee's, suggesting greater diversity. This increased diversity appears to be mainly due to a higher number of nonsynonymous SNPs, since the chimpanzee's synonymous θ value is higher than that of human's. These results lend support to an increased level of divergence, especially at nonsynonymous sites, occurring in the human lineage. However, such a disparity between these species is seen often, due to the recent population explosion in humans.

From these results we conclude that *KLF14* is a highly variable gene relative to other genes in the genome, and its variability is not common to chimpanzees. This high level of variability lends support to the finding that *KLF14* has undergone, and may be continuing to undergo, accelerated evolution in the human lineage.

## Discussion

### Imprinting of *KLF14*

We have identified a novel imprinted gene, *KLF14*, and show that it is maternally expressed in every tissue examined in both human and mouse. The gene encodes a putative Krüppel-like transcription factor, containing three C2H2 zinc-fingers joined together by a characteristic linker sequence [19]. The function of KLF14 is currently unknown. Previous studies have shown that it is similar to KLF16 [36], and an alignment of the two proteins identified a conserved α-helical repression motif at the N terminus, which has been shown to act as a transcriptional repressor by directly interacting with mSin3A-histone deacetylase [51,52], suggesting that KLF14 may also share this regulatory activity.

The relatively greater expression level of *KLF14* in embryonic and extra-embryonic tissues compared to adult tissues suggests a role for the transcript in development. Indeed, many imprinted genes regulate growth and embryonic development (for a review, see [30]). This observation has led to the proposal of a conflict hypothesis, where maternally expressed genes suppress fetal growth, allowing nutrient supply to be available for future pregnancies and increasing the mother's survival rate. In contrast, paternally expressed transcripts enhance fetal growth to ensure survival of their genetic offspring [53]. Under this hypothesis, the maternally expressed *KLF14* is predicted to suppress embryonic growth. This, together with its predicted function as a transcriptional repressor, suggests that the protein may suppress the expression of genes that enhance fetal or placental development.

Our analysis of the *Klf14* CpG island indicates that the region is hypomethylated. The identification of an unmethylated CpG-rich region in an imprinted cluster has been previously described, such as the promoter region of *Dlk1* [54], the promoter and first exon of Gsα [55], and *Ascl2* [56]. Our ChIP results, performed using antibodies specific to various histone modifications, did not identify differences between the two alleles of *Klf14*. However, clear allele-specific precipitation of histone modifications was observed at the DMR of *Mest*. This is the first description of allele-specific histone modifications associated with the *Mest* germ-line DMR. Despite the lack of these trademark epigenetic features at the *Klf14* CpG island, its imprinting is maintained, and its expression is dependent on the function of *Dnmt3a* in female germ cells. To date, the only DMR identified in this region is the maternally methylated CpG island at the 5′ end of *MEST/Mest,* which has been shown to be established in gametes [32]. As such, we propose that this DMR may regulate the imprinted expression of *KLF14,* possibly through long-range chromatin regulatory interactions and may act as an ICR for the entire locus, spanning carboxypeptidase-A4 *(CPA4)* to *KLF14* (Figure 3E). Further studies are necessary to determine if the expression of these genes is regulated by long-range insulator or enhancer elements, as has been shown for genes in the *H19*/*Igf2* locus and *KCNQ1* region [57].

### Evolution of *KLF14*

Our analysis suggests that *KLF14* arose through the retrotransposition of *KLF16*. Thus, *KLF14* is the ninth imprinted retrotransposed gene identified to date and the first protein-coding maternally expressed retrotransposed gene identified in mouse, adding further support to the hypothesis that imprinting serves as a mechanism for regulating increased gene dosage [1]. We postulate that *KLF14* acquired imprinting through *cis-* and *trans-*acting elements associated with the more ancient *MEST,* which is known to be imprinted in marsupials [58]. This would allow the host eutherian mammal to adapt to the increased gene dosage caused by the retrotransposition of *KLF16*. By analyzing the expression and epigenetic modifications of *KLF14* and other retrotransposed genes in eutherian mammals, it may be possible to elucidate the mechanism whereby these genes have acquired imprinted expression and the control elements upon which their imprinting depend.

*KLF14*, unlike its closest relatives in the KLF family of genes, has a large CpG island spanning the vast majority of its ORF. At the same time, the gene is enriched for proline and arginine amino acids, which are encoded by the codons CCN and CGN, respectively. Hence, it is plausible that, upon the retrotransposition of *KLF16,* the fusion of the proline/arginine-rich exons created a CG-rich region, which is predicted to be a CpG island, yet lacks the biological functions generally associated with such regions. Such an occurrence would also account for the absence of epigenetic modifications observed in the CpG island. Thus, we cannot exclude the existence of a CG-neutral differentially methylated promoter or additional exons upstream of the *KLF14* ORF. However, attempts to identify the 5′′ end of the gene in both human and mouse were fruitless (unpublished data).

The variability observed in the *KLF14* ORF in humans, particularly the three fixed nonsynonymous changes and two nonsynonymous SNPs with higher frequencies of the derived allele, are suggestive of positive selection. A recent investigation of selection in the human and chimpanzee genomes identified *KLF14* as a gene that has undergone adaptive evolution in the human genome [59]. However, our detailed analyses of this gene failed to provide significant results in tests for positive selection, even when the gene was divided into the variable N terminus and the conserved C-terminal zinc-finger domains (unpublished data). Since the null hypothesis of neutrality could not be rejected, the evolution observed in *KLF14* could be due to relaxed functional constraint. The lack of significance observed may be caused by limitations in the analyses due to the gene's small size or by ongoing positive selection, as observed in previous studies [60]. Evidence for the latter lies in the numerous polymorphisms that are not fixed in the human lineage. Further experiments, such as the Extended Haplotype Homozygosity method [60] are needed to elucidate whether selection is active at this locus.

While we cannot reject neutrality or provide conclusive evidence that *KLF14* is under positive selection, we demonstrate that this gene has undergone accelerated evolution in the human lineage. It is important to note that although the dN/dS values corresponding to the human lineage are >1, this accelerated evolution may in fact be caused by a relaxation of purifying selection. Although such a relaxation is unlikely, due to *KLF14*'s conservation throughout mammalian evolution, the gene's functional significance may have changed or decreased within the human lineage, allowing for multiple mutations to accumulate in nonsynonymous sites.

Numerous studies have focused their efforts on the identification of genes under positive selection or accelerated evolution in the human species [61–63] and have found that several functional categories, including transcription factors, are enriched for rapidly evolving transcripts [62]. Since *KLF14* had not been fully sequenced in the chimpanzee, and possibly due to the gene's size, these studies did not detect positive selection in the transcript. Thus, our analysis of *KLF14* adds to the mounting body of literature suggesting that transcription factors are often evolving rapidly or with positive selection. However, *KLF14* stands alone in this category due to its imprinting, which may have played a role in the evolution of this transcript. Previous papers have also failed to find evidence of positive selection across imprinted genes [64,65].

Our data suggest that the *KLF14* sequence is highly variable, specifically in the human species. We propose that this variability may be due to the transcript's monoallelic expression, which allows for the accumulation of mutations on the silenced allele. Maternal inheritance of mutated alleles would give rise to their expression and deleterious mutations would face strong purifying selection due to haploinsufficiency. In contrast, beneficial mutations would undergo stronger and more rapid positive selection since their impact would be greater due to the gene's monoallelic expression. The latter phenomenon has been implicated in the increased rate of nonsynonymous substitution on the X chromosome [66]. Consequently, the inherited variations seen in *KLF14* should be nondeleterious and possibly advantageous. This is supported by the fact that the haplotypes carrying rare alleles are transmitted from both mothers and fathers and are consequently expressed in healthy individuals, as evidenced in the unaffected siblings of RSS and autism patients. All of these sequence variations, with the exception of a synonymous polymorphism observed in haplotype 6 (Figure S1), occur in the N-terminal end of the putative protein that has low sequence conservation, suggesting that variation in the C-terminal end of the protein may not be tolerated.

In 2004, Dorus and colleagues examined the evolutionary rates of nervous system-related genes between primates and rodents, thereby identifying genes under accelerated evolution in primates. They proposed that these genes may have played important roles in human speciation by developing human behavior and brain size [61]. Consequently, the disruption of genes under accelerated evolution or positive selection in the human lineage has been associated with disease. For example, mutations in *FOXP2* underlie severe language and speech impairment/developmental verbal dyspraxia [5,67], while *Microcephalin* and *ASPM* are associated with microcephaly [68,69]. Due to *KLF14*'s increased expression in neuronal cells, as well as the accelerated evolution observed in the human lineage, this gene may have played a role in the acquisition of human-specific traits. Such a function would agree with our hypothesis postulated above, describing the role of imprinting in the variability of the gene. Despite being imprinted in ancestral mammals, we observe that the evolutionary aspects of *KLF14* are unique to the human lineage. This observation could be due to selective pressures unique to this species, and possibly unique to demographic populations within the human population, which have accelerated the evolution of the gene. Consequently, the variations seen in *KLF14* may be beneficial to humans or subpopulations of the human species, particularly the variations that are fixed or are going towards fixation. However, further studies are required to determine the gene's function in the brain, particularly in neurons, to assess its contribution to human speciation and its putative role in cognitive disease.

Although, the sequencing of the *KLF14* ORF in autistic individuals and RSS patients did not identify mutations unique to these populations, it does not rule out the involvement of *KLF14* in these phenotypes since mutations may be present in regulatory regions causing changes in expression levels, loss of imprinting, or transcript instability. Due to the lack of *KLF14* expression in lymphoblasts, we were unable to quantify transcript levels in patients, nor were we able to verify imprinted expression in these patients. Future studies may ascertain the involvement of *KLF14* in these disorders by obtaining fibroblast cells from patients, thereby elucidating the role of both imprinted genes and positively selected genes on development and cognitive function, respectively.

## Materials and Methods

### RT-PCR using RNA from somatic cell hybrids, human tissues, and murine embryonic samples.

cDNA was obtained and amplified from somatic cell hybrids–cell lines and human tissues [22]. The primers used for amplification of *KLF14* are forward (5′-CCACCCAACCTATCATCCAG-3′) and reverse (5′-GTACCTCCCCAGAGTCCACA-′). Reciprocal hybrid crosses were performed between C57BL/6J and JF1/Ms, and tissues were obtained from the *F*_1_ generations. Glia and neurons were cultured as previously described [34], and cDNA was obtained [22]. To identify genomic SNP in *Klf14,* an 851-bp fragment was amplified using primers AK030435F (5′-TGGACACCCTCTCCAAAGTC-3′) and AK030435R (5′-AAGCGACATCAGTGCTCCTT-3′), and a SNP corresponding to bp 451 of AK030435 was found. Amplified DNA and cDNA fragments were purified and directly sequenced.

### Methylation analysis.

Genomic DNA was extracted from BL6 × JF1 12.5-dpc whole embryos and fibroblasts. Bisulfite treatment was performed using the EZ methylation protocol (Zymo Research, http://www.zymoresearch.com). One microliter of the eluted DNA was used for PCR using the following primers: *F*_1_, 5′-TGGTTGTAATAAGGTTTATTATAAGT-3′ and *R*_1_, 5′-AAACCAAAACTTTCCACCATAACTA-3′; *F*_2_, 5′-TGGAGGATTGGGGGTATTTATA-3′ and *R*_2_ 5′-CAAACAAATAATTTCCCAAACTACTAA-3′; *F*_3_ 5′-TTTGGGGTTATTTTTTATTTGAGTT-3′ and *R*_3_ 5′-TCAAACAAAATCCTAAAAACTTTTT-3′. PCR products were subcloned using the pGEM-T easy system (Promega, http://www.promega.com), and transformants were sequenced.

### RACE to determine full-length sequence of *KLF14.*

Marathon ready cDNA from 11-day embryo and placenta (Clontech, http://www.clontech.com) were used for RACE to determine the full length of *KLF14* in mouse and human, respectively. The manufacturer's protocol was followed using the following primers for 3′ RACE: human GSP1 (5′- GAAGGGATGAACTCCCGTACTCTCCA-3′), human GSP1-nested (5′-AACCAGGGATGTGAAACTGG-3′), mouse GSP1 (5′-GGGTGTTGTGATCTCATGGAGTTG-3′), and mouse GSP1-nested (5′-TGCTAAGTTTCTGCCAAGAGC-3′). The amplified cDNA fragments were purified using microCLEAN (Microzone, http://www.microzone.co.uk) and directly sequenced.

### ChIP and analysis of histone modification.

Formaldehyde fixed ChIP assay was carried out using Chromatin Immunoprecipitation Assay Kit (Upstate Biotechnology, http://www.upstate.com) as previously described [70]. The antisera used were against H3K9acK14ac (Upstate Biotechnology, 06–599), H4ac (Upstate Biotechnology, 06–866), and H3K4me2 (Upstate Biotechnology, 07–030). DNA obtained from the precipitated fractions was amplified using primers for *Mest* (MCF1: 5′-AGGGGGTAGCGGGTCAATAC-3′ and MCR1: 5′-ATGTGCTGGTGGCCGAAGCAG-3′) and *Klf14* (KCF1: 5′-TTGGAGCCAGACGAGCTGGAAG-3′ and KCR1: 5′-AGGCTGCTGGGAATGCCATAGC-3′). Alleles between BL6 and JF1 were distinguished by *Mae*I and *Sac*II polymorphisms. We determined the dominant alleles by analyzing band intensities.

At the same time, ChIP was performed using nonfixed chromatin from BL6 × JF1 13.5-dpc hybrid embryos, and precipitated DNA was analyzed by PCR single strand conformation polymorphism as previously described [71]. The antibodies used for native ChIP were directed against H3K9ac (Upstate Biotechnology, 06–942), H3K4me2 (Upstate Biotechnology, 07–030), H3K9me3 (Upstate Biotechnology, 07–442), and H4K20me3 (Upstate Biotechnology, 07–463).

### Amplification of *KLF14* and *KLF16* in mammalian species.

The *KLF14* ORF was sequenced in 352 individuals (704 chromosomes), representing both patients (60 RSS and 160 autistic) and controls (78 individuals of Western European descent from the human variation panel and 54 individuals from an ethnically diverse panel containing nine African American, four Arabian, four Armenian, four Chinese, three Greek, four Indo-Pakistani, four Italian, four Iranian, eight Japanese, and 10 Somalian individuals). Haplotypes were determined manually, and when necessary, samples were subcloned to determine the phase of polymorphisms. The *KLF14* ORF was also sequenced from three gorillas, two orang-utans, two macaques, two bonobos, and 20 chimpanzees. In the latter, at least one individual from three chimpanzee subspecies was included.

Primers used to amplify KLF16 were: *F*_1_ (5′-CCCGCCACCACCGGAC-3′) or *F*_2_ (5′-CCCGGCACTACCGGAC-3′) and *R* (5′-TGCAGGGCAGCGAGTCG-3′). KLF14 was amplified using the following primers and combinations thereof: *F*_1_ (5′-AACTTCTTGTCGCAGTCGAG-3′), *F*_2_ (5′-ACTTCTTGTCGCAGTCGA-3′), *R*_1_ (5′-CGTGCCTGGACTACTTCGC-3′), *R*_2_ (5′-GCCCCACCTGCTGGCT-3′), *R*_3_ (5′-GCCCCACCTGCTGGC-3′).

### Evolution analyses of *KLF14.*

Primate and human haplotype sequences were manually aligned and used to create a parsimony tree (Figure 5B). This tree does not represent the evolution of the gene, since haplotypes evolve through recombination as well as mutation making phylogeny difficult to reconstruct. Instead, the tree represents the similarity between the sequences. A neighbor-joining tree was also created from this alignment using MEGA3 [72]. We created nine other joining-joining primate trees, with each one using a single human haplotype or the HH-MRCA, created manually by parsimony, as the human sequence. These trees were used to test the neutral model of molecular evolution within the primate lineages. Lineage-specific dN/dS ratios were computed using codon-based maximum likelihood methods. These dN/dS tests were performed using the CodeML program within the PAML software package [73], which uses the codon substitution model of Goldman and Yang [74]. In these models we allowed for unequal codon frequencies and unequal transition and transversion substitution rates.

The lineage-specific dN/dS ratios for each of the nine single human haplotype trees were calculated using the maximum likelihood free-ratio model, as well as the Nei-Gojobori method (Figure 5B) [75]. The likelihood values and dN/dS ratios for each tree were also calculated, using the one-ratio model, the two-ratio model, where the ratio of the human lineage is allowed to differ from the ratio of the other primate lineages, and the two-ratio model, with dN/dS of the human lineage set equal to one. To test the null hypothesis that *KLF14* is evolving neutrally in the human lineage, the likelihood ratio test was used (Table S1) [39] to assess whether *KLF14*'s human dN/dS ratio is significantly greater than one or if the human dN/dS ratio is significantly greater than the primate background.

Additionally, 9,880 bps (including the ORF) encompassing *KLF14* were sequenced in 18 individuals from the diversity panel and in one chimpanzee (to deduce the ancestral allele). Haplotypes were inferred using the PHASE program [76], and DnaSP 4.0 [40] was used to test for positive selection using the Tajima's D-test [41], Fu and Li's D-test [42], Fu and Li's F* [42], Fu's Fs statistic [43], and Fay and Wu's H test statistic [44].

To detect positive selection, the McDonald-Kreitman test [45] was performed using the ORF sequences from the 20 chimpanzee and two bonobo samples. DnaSP 4.0 [40] was then used to look for a significant departure from neutrality. A binomial distribution test [46] was also used to look for an increased rate of amino acid substitutions in the human lineage relative to the ape lineages, which would suggest that accelerated amino acid evolution has occurred in the human lineage.

### Sequence variability in *KLF14.*

Polymorphism data were extracted on 826 genes sequenced in the Seattle SNP and Environmental Genome (EG) projects. π values were calculated for each of the sequenced genes and for *KLF14* using the allele frequencies for each identified polymorphism. Values of π were calculated using SNPs at all sites, at sites with synonymous polymorphisms, and at sites with nonsynonymous polymorphisms. Diversity statistics π and θ were also calculated for both the human and chimpanzee *KLF14* gene to compare the variability between species.

## Supporting Information

Figure S1 *KLF14* ORF Sequences in the Human, Chimpanzee, and Gorilla Shown are eight different haplotypes (1–6, 7J, and 8J) identified in diverse human populations, as well as the sequence for the *KLF14* ORF in the chimpanzee and gorilla (C and G, respectively). Synonymous polymorphisms and nonsynonymous polymorphisms are denoted by gray and black boxes, respectively. The corresponding amino acid substitution is noted above each polymorphism. The identity of the ancestral allele was identified by comparison to orang-utan (Pongo pygmaeus) and macaque (Macaca mulatta). The sequence corresponding to zinc-finger domains are enclosed, and the sequence between the boxes comprises the conserved Krüppel-link. Haplotypes specific to the Japanese population are indicated by J.
(563 KB PDF)

Table S1 Likelihood Ratio Tests for Positive Selection and Accelerated Evolution (52 KB DOC)

Table S2 Acceleration Index Test Using Binomial Distribution (35 KB DOC)

Table S3 Diversity Statistics for Human and Chimpanzee (25 KB DOC)

Table S4 Chimpanzee and Bonobo Genotypes (53KB DOC)

Table S5 Gorilla Genotypes (27 KB DOC)

Text S1 Supplementary Data (31 KB DOC)

### Accession Numbers

The Online Mendelian Inheritance in Man (OMIM) (http://www.ncbi.nlm.nih.gov/entrez/query.fcgi?db=OMIM) numbers discussed in this paper are Prader-Willi syndrome, MIM:176270; Beckwith-Wiedemann syndrome, MIM:130650; and RSS, MIM:180860. The National Center for Biotechnology Information (NCBI) (http://www.ncbi.nlm.nih.gov) accession numbers for the genes and gene products discussed in this paper are *KLF14,* NM_138693; *Klf14,* AK030435 and DQ534758; and *KLF16,* NP_114124.

## Abbreviations

ChIP: chromatin immunoprecipitation
DMR: differentially methylated region
dN/dS: ratio of nonsynonymous to synonymous substitutions
*Dnmt3a*: DNA methyltransferase 3a
dpc: days post coitum
H3K4me2: dimethylation of lysine 4 on histone 3
H3K9ac: acetylation of lysine 9 on histone 3
H3K9me3: trimethylation of lysine 9 on histone 3
H4K20me3: trimethylation of lysine 20 on histone 4
HH-MRCA: human haplotype's most recent common ancestor
ICR: imprinting control region
*KLF14*: Krüppel-like factor 14
*MEST*: mesoderm specific transcript homolog
MRCA: most recent common ancestor
ORF: open reading frame
PAML: phylogenetic analysis by maximum likelihood
RACE: rapid amplification of cDNA ends
RSS: Russell-Silver Syndrome
RT-PCR: reverse transcriptase-PCR
SNP: single nucleotide polymorphism
