# Supplementary material for: Identification of the Imprinted KLF14 Transcription Factor Undergoing Human-Specific Accelerated Evolution
Source: PLoS Genet. 2007 May 4;3(5):e65. doi: 10.1371/journal.pgen.0030065 (PMC1865561; doi:10.1371/journal.pgen.0030065)
Supplement: Supplementary file 2 [file pgen.0030065.sd001.doc]

**Text S1.**

**Supplementary Data**

**Additional Evolutionary Analyses of *KLF14***

In addition to the pairwise (single human haplotype versus primates) analyses presented in the Results, we simultaneously investigated whether there was significant elevation in the human dN/dS using all of the human haplotypes. For this analysis we created a haplotype tree using the neighbour-joining method within MEGA3 [72]. One caveat is that this haplotype tree does not represent the true evolution of the haplotypes. Due to the diploid nature of DNA, haplotypes often evolve by recombination, as well as mutation. As a result, the tree constructed by this analysis is a similarity tree, which we assumed as a “best guess” for the evolution of the haplotypes. We used PAML [39] to investigate whether there is evidence for positive selection in the branches of the haplotype tree. Multiple two-ratio and three-ratio tests were performed (data not shown). Although some lineages had dN/dS values that suggested positive selection, they were not significantly >1 when tested using the likelihood ratio test.

We also used PAML to look for positive selection at individual amino acid sites within specific lineages of the human haplotype tree [77,78]. Multiple models that look for variable dN/dS ratios among sites within lineages did not detect sites that showed significant evidence for positive selection using the likelihood ratio test. Thus, our extensive searches for positive selection using PAML’s maximum likelihood methods did not yield significant results. We are therefore unable to reject the hypothesis of neutral evolution in the human lineage.
