# Supplementary material for: Identification of the Imprinted KLF14 Transcription Factor Undergoing Human-Specific Accelerated Evolution
Source: PLoS Genet. 2007 May 4;3(5):e65. doi: 10.1371/journal.pgen.0030065 (PMC1865561; doi:10.1371/journal.pgen.0030065)
Supplement: Supplementary file 3 [file pgen.0030065.st001.doc]

**Supplementary Table 1:** Likelihood Ratio Tests for Positive Selection and Accelerated Evolution

| **Haplotype** | **lnL (one-ratio)** | **lnL (two-ratio; ω2=1)** | **lnL (two-ratio)** |
| --- | --- | --- | --- |
| HH-MRCA^a^ | -1560.286661 | -1558.381166 | -1557.261724 |
| 1 | -1579.420414 | -1577.087192 | -1576.757330 |
| 2 | -1574.548406 | -1571.579565 | -1569.719047 |
| 3 | -1567.082891 | -1565.859071 | -1565.825299 |
| 4 | -1580.904547 | -1578.565884 | -1578.249591 |
| 5 | -1580.907174 | -1578.568019 | -1578.251491 |
| 6 | -1580.904241 | -1578.565351 | -1578.248966 |
| 7 | -1580.524455 | -1577.087817 | -1574.847433 |
| 8 | -1586.774089 | -1583.931347 | -1583.404248 |

One-ratio VS Two-ratio with ω2=1 (test for dN/dS significantly greater than 1; test for positive selection)

| **Haplotype** | **2Δl** | **df** | **P-value** | **P-value (Bonferroni)** |
| --- | --- | --- | --- | --- |
| HH-MRCA^a^ | 2.238884 | 1 | 0.1345 |  |
| 1 | 0.659724 | 1 | 0.4166 | 1.0000 |
| 2 | 3.721036 | 1 | 0.0537 | 0.4296 |
| 3 | 0.067544 | 1 | 0.7949 | 1.0000 |
| 4 | 0.632586 | 1 | 0.4264 | 1.0000 |
| 5 | 0.633056 | 1 | 0.4262 | 1.0000 |
| 6 | 0.632770 | 1 | 0.4263 | 1.0000 |
| 7 | 4.480768 | 1 | 0.0342* | 0.2736 |
| 8 | 1.054198 | 1 | 0.3045 | 1.0000 |

One-ratio VS Two-ratio (test for dN/dS significantly greater than background; test for accelerated evolution)

| **Haplotype** | **2Δl** | **df** | **P-value** | **P-value (Bonferroni)** |
| --- | --- | --- | --- | --- |
| HH-MRCA^a^ | 6.049874 | 1 | 0.0139* |  |
| 1 | 5.326168 | 1 | 0.0210* | 0.1680 |
| 2 | 9.658718 | 1 | 0.0018** | 0.0144* |
| 3 | 2.515184 | 1 | 0.1127 | 0.9016 |
| 4 | 5.309912 | 1 | 0.0212* | 0.1696 |
| 5 | 5.311366 | 1 | 0.0211* | 0.1688 |
| 6 | 5.310550 | 1 | 0.0211* | 0.1688 |
| 7 | 11.354044 | 1 | 0.0007** | 0.0056** |
| 8 | 6.739682 | 1 | 0.0094** | 0.0752 |

^a^ human haplotype’s most recent common ancestor

* P < 5%

** P < 1%
