# Supplementary material for: Identification of the Imprinted KLF14 Transcription Factor Undergoing Human-Specific Accelerated Evolution
Source: PLoS Genet. 2007 May 4;3(5):e65. doi: 10.1371/journal.pgen.0030065 (PMC1865561; doi:10.1371/journal.pgen.0030065)
Supplement: Supplementary file 4 [file pgen.0030065.st002.doc]

**Supplementary Table 2**: Acceleration Index Test Using Binomial Distribution

| **Haplotype** | **n-Human** | **n-Ape** | **Expected Proportion** | **Tail Probabilty** | **Bonferroni Correction** | |
| --- | --- | --- | --- | --- | --- | --- |
| HH-MRCA^a^ | 3 | 4 | 0.13268 | 0.054020 |  |  |
| 1 | 5 | 4 | 0.13268 | 0.003251 | 0.026008 | * |
| 2 | 5 | 4 | 0.13268 | 0.003251 | 0.026008 | * |
| 3 | 3 | 4 | 0.13268 | 0.054020 | 0.432160 |  |
| 4 | 5 | 4 | 0.13268 | 0.003251 | 0.026008 | * |
| 5 | 5 | 4 | 0.13268 | 0.003251 | 0.026008 | * |
| 6 | 5 | 4 | 0.13268 | 0.003251 | 0.026008 | * |
| 7 | 6 | 4 | 0.13268 | 0.000708 | 0.005664 | ** |
| 8 | 6 | 4 | 0.13268 | 0.000708 | 0.005664 | ** |

^a^ Human haplotype’s most recent common ancestor

* P < 5%

** P < 1%
