# Supplementary material for: Identification of the Imprinted KLF14 Transcription Factor Undergoing Human-Specific Accelerated Evolution
Source: PLoS Genet. 2007 May 4;3(5):e65. doi: 10.1371/journal.pgen.0030065 (PMC1865561; doi:10.1371/journal.pgen.0030065)
Supplement: Supplementary file 5 [file pgen.0030065.st003.doc]

**Supplementary Table 3:** Diversity Statistics for Human and Chimpanzee

|  | **# of chromosomes** | **Total Gene (966 sites)** | | | **Nonsynonymous (703 sites)** | | | **Synonymous (263 sites)** | | |
| --- | --- | --- | --- | --- | --- | --- | --- | --- | --- | --- |
|  |  | **SNPs** | **Pi (%)** | **Theta (%)** | **SNPs** | **Pi (%)** | **Theta (%)** | **SNPs** | **Pi (%)** | **Theta (%)** |
| **Human** | 264 | 10 | 0.168 | 0.168 | 6 | 0.108 | 0.139 | 4 | 0.326 | 0.247 |
| **Chimpanzee** | 40 | 3 | 0.016 | 0.073 | 0 | 0 | 0 | 3 | 0.057 | 0.268 |
