# Supplementary material for: Identification of the Imprinted KLF14 Transcription Factor Undergoing Human-Specific Accelerated Evolution
Source: PLoS Genet. 2007 May 4;3(5):e65. doi: 10.1371/journal.pgen.0030065 (PMC1865561; doi:10.1371/journal.pgen.0030065)
Supplement: Supplementary file 6 [file pgen.0030065.st004.doc]

**Supplementary Table 4:** Chimpanzee and Bonobo genotypes

| **ID** | **Species** | **IPBIR No.** | **ISIS No.** | **ss60197533** | **ss60197534** | **ss60197535** |
| --- | --- | --- | --- | --- | --- | --- |
| Pt82 | *P. troglodytes verus* | - |  | G/G | C/C | G/G |
| Pt87 | *P. troglodytes verus* | - | 1149 | G/G | C/C | G/G |
| Pt90 | *P. troglodytes verus* | - | 1339 | G/G | C/C | G/G |
| Pt91 | *P. troglodytes verus* | - | 1506 | G/G | C/C | G/G |
| Pt96 | *P. troglodytes schweinfurthii* | - | 3020 | G/A | C/A | G/G |
| Pt97 | *P. troglodytes verus* | - | 2036 | G/G | C/C | G/G |
| Pt98 | *P. troglodytes verus* | - | 2724 | G/G | C/C | G/G |
| Pt100 | *P. troglodytes verus* | - | 3000 | G/G | C/C | G/G |
| Pt104 | *P. troglodytes verus* | - | 3339 | G/G | C/C | G/G |
| Pt105 | *P. troglodytes verus* | - | 2435 | G/G | C/C | G/G |
| Pt117 | *P. troglodytes verus* | - | 1641 | G/G | C/C | G/G |
| Pt122 | *P. troglodytes verus* | - | 5417 | G/G | C/C | G/G |
| Pt124 | *P. troglodytes verus* | - | 2404 | G/G | C/C | G/G |
| Pt161 | *P. troglodytes schweinfurthii* | - | 925 | G/G | C/C | G/G |
| Pt196 | *P. troglodytes verus* | PR00226 | 2000 | G/G | C/C | G/G |
| Pt197 | *P. troglodytes troglodytes* | PR00496 | 351 | G/G | C/C | G/A |
| Pt198 | *P. troglodytes verus* | S006006 | - | G/G | C/C | G/G |
| Pp14 | *P. paniscus* | PR00367 | A01013 | G/G | C/C | G/G |
| Pp15 | *P. paniscus* | PR00092 | 593203 | G/G | C/C | G/G |
| Pp16 | *P. paniscus* | PR00251 | 691777 | G/G | C/C | G/G |
