# Supplementary material for: Identification of the Imprinted KLF14 Transcription Factor Undergoing Human-Specific Accelerated Evolution
Source: PLoS Genet. 2007 May 4;3(5):e65. doi: 10.1371/journal.pgen.0030065 (PMC1865561; doi:10.1371/journal.pgen.0030065)
Supplement: Supplementary file 7 [file pgen.0030065.st005.doc]

**Supplementary Table 5: Gorilla genotypes**

| **ID** | **Species** | **IPBIR No.** | **ISIS No.** | **ss60197531** | **ss60197532** |
| --- | --- | --- | --- | --- | --- |
| Ggo7 | *Gorilla gorilla* | PR00107 | 1890 | C/C | C/C |
| Ggo8 | *Gorilla gorilla* | PR00573 | 27944 | G/G | C/C |
| GL3-05 | *Gorilla gorilla* | - | - | G/G | C/A |
